# Combined elevation of myeloid-derived suppressor cells and Tregs predicts lymph node metastasis in breast cancer

**DOI:** 10.1186/s12885-025-15277-4

**Published:** 2025-11-24

**Authors:** Huichao Zhang, Xiaojie Yin, Sifan Wang, Qingjing Li, Longfei Xia, Jingfu Qiu, Wei Li, Hong Li, Feng Zhao, Yuan Song

**Affiliations:** 1 Department of Clinical Laboratory, The Fourth Hospital of Hebei Medical University, No.12, JianKang Road, Shijiazhuang, Hebei 050000 China; 2 Department of Clinical Laboratory, Zibo Central Hospital, Zibo Shandong, 255020 China; 3 Department of Health Laboratory Technology, School of Public Health, Chongqing Medical University, Chongqing, 400016 China; 4 Hebei Junlin Pharmaceutical Co., LTD, Xingtai, Hebei 054300 China

**Keywords:** Breast cancer, MDSCs, PMN-MDSCs, M-MDSCs, Tregs, ROC curve analysis

## Abstract

**Background:**

This study investigates the clinical significance of myeloid-derived suppressor cells (MDSCs), including polymorphonuclear MDSCs (PMN-MDSCs) and monocytic MDSCs (M-MDSCs) subsets, and regulatory T cells (Tregs) in the peripheral blood of breast cancer patients.

**Methods:**

Using flow cytometry, we analyzed levels of these immunosuppressive cell populations in 107 breast cancer patients and 33 healthy controls at the Breast Centre of the Fourth Hospital of Hebei Medical University (July-November 2021).

**Results:**

Levels of MDSCs, PMN-MDSCs, M-MDSCs, and Tregs were significantly elevated in breast cancer patients compared to controls (*P* < 0.05). Furthermore, MDSCs, PMN-MDSCs, and Tregs showed a strong positive correlation with lymph node metastasis (*P* < 0.001). M-MDSCs were also significantly associated, though to a lesser extent (*P* = 0.045). Receiver operating characteristic (ROC) curve analysis revealed high diagnostic value for these cells in breast cancer. Notably, Tregs exhibited the highest individual area under the curve (AUC = 0.766) for detecting lymph node metastasis. Critically, the combined assessment of MDSCs and Tregs significantly enhanced predictive accuracy for lymph node metastasis, yielding a combined AUC exceeding that of any single marker.

**Conclusion:**

Elevated levels of MDSCs (and their subsets) and Tregs are closely associated with lymph node metastasis in breast cancer. Their combined detection provides an effective predictive tool for lymph node involvement and holds substantial clinical value.

## Introduction

 Breast cancer remains a leading cause of cancer-related morbidity and mortality in women worldwide [1, 2]. Myeloid-derived suppressor cells (MDSCs) are key drivers of immune dysregulation, promoting breast cancer progression and metastasis [3]. Their levels in blood are closely linked to advanced disease stage and metastatic burden. Furthermore, specific MDSCs subtypes have distinct clinical associations: polymorphonuclear MDSCs (PMN-MDSCs) with triple-negative breast cancer, and monocytic MDSCs (M-MDSCs) with estrogen receptor (ER)-negative status and liver or bone metastasis [3]. These findings position MDSCs as pivotal contributors to breast cancer pathogenesis and promising candidates for biomarker development and therapeutic targeting.

MDSCs are a heterogeneous group of immature bone marrow-derived myeloid cells with significant immunosuppressive properties [4, 5]. Under normal physiological conditions, MDSCs differentiates into granulocytes, dendritic cells and macrophages, which are involved in immune regulation. However, in pathological conditions such as cancer, chronic inflammation and infection, these cells proliferate abnormally and suppress the immune response [6].

The mechanisms underpinning MDSCs-mediated immune evasion are multifaceted. MDSCs suppress immunity by inhibiting T and Natural Killer cell activation, promoting T helper 17 (Th17) differentiation, and expanding regulatory T cells (Tregs) [7]. They achieve this by secreting factors like arginase-1 (Arg-1) and inducible nitric oxide synthase (iNOS), which deplete amino acids, generate reactive oxygen and nitrogen species (ROS/RNS), and ultimately disrupt lymphocyte function [8]. Furthermore, MDSCs disrupt immune cell trafficking and differentiation, fostering an immunosuppressive tumour microenvironment (TME) conducive to Tregs expansion [8–10]. This synergy between MDSCs and Tregs establishes a formidable barrier to anti-tumour immunity. Consequently, elevated MDSCs levels are strongly linked to tumour progression, metastasis, diminished patient survival, and resistance to immunotherapy [6, 10]. Beyond immunosuppression, MDSCs actively fuel tumour growth by promoting angiogenesis, invasion, and the establishment of pre-metastatic niches. Targeting MDSCs thus represents a compelling strategy to restore immune surveillance and improve therapeutic outcomes, potentially halting disease progression.

Building on this foundation, our study specifically investigates the levels and clinical significance of circulating MDSCs (and their PMN-MDSCs and M-MDSCs subsets) and Tregs in breast cancer patients. We hypothesize that coordinated elevations in these immunosuppressive populations are linked to lymph node metastasis, a critical determinant of prognosis and treatment. By elucidating the interplay and diagnostic potential of MDSCs and Tregs, we aim to identify novel combinatorial biomarkers and inform the development of more effective diagnostic and therapeutic strategies for breast cancer and other malignancies.

## Materials and methods

### Study population and design

We conducted a prospective observational study at the Breast Centre of the Fourth Hospital of Hebei Medical University between July 2021 and November 2021. A total of 253 consecutive female patients newly diagnosed with breast cancer were initially screened. After applying strict inclusion and exclusion criteria, 107 patients formed the final breast cancer cohort. Thirty-three age-comparable healthy female volunteers undergoing routine health check-ups at the same institution between October 12 and November 2, 2021, served as controls. Controls were excluded if they had any history of malignancy, autoimmune disease, active cardiovascular disease, acute or chronic infection, or were receiving immunosuppressive therapy.

### Inclusion and exclusion criteria

 Inclusion criteria: (1) Histopathologically confirmed diagnosis of breast cancer according to the 2018 Chinese Society of Clinical Oncology Breast Cancer Guidelines; (2) Complete clinicopathological data available; (3) Complete laboratory test results (including flow cytometry) available. Exclusion criteria: (1) Incomplete clinical data (*n* = 67 excluded); (2) Incomplete or missing laboratory data (*n* = 72 excluded); (3) Diagnosis of rare pathological subtypes (e.g., ductal carcinoma in situ, invasive lobular carcinoma, invasive micropapillary carcinoma) where sample size was insufficient for meaningful statistical analysis (*n* = 7 excluded). Note: Patients could meet multiple exclusion criteria. The final analysis cohort comprised 107 breast cancer patients. All relevant clinical characteristics of the patient and control groups are summarized in Table 1.

**Table 1:** Subgroup analysis of MDSCs, PMN-MDSCs, M-MDSCs, and Tregs in breast cancer patients. Values expressed as median (IQR). *P < 0.05; **P < 0.01; ***P < 0.001

Clinical indicator		n (%)	MDSCs		PMN-MDSCs		M-MDSCs		Tregs	
			Median(IQR)	*P*	Median(IQR)	*P*	Median(IQR)	*P*	Median(IQR)	*P*
Age	< 50	47(43.9)	0.100(0.13)	0.232	0.040(0.05)	0.199	0.030(0.10)	0.347	2.950(2.54)	0.296
	> 50	60(56.1)	0.110(0.15)		0.050(0.07)		0.040(0.09)		3.590(2.43)	
Menstrual state	menstrual	60(56.1)	0.105(0.15)	0.398	0.050(0.06)	0.255	0.040(0.10)	0.405	3.590(2.42)	0.178
	pre-menstrual	47(43.9)	0.100(0.13)		0.040(0.05)		0.030(0.09)		3.080(2.30)	
Clinical staging	N0	54(50.5)	0.080(0.10)	<0.001***	0.030(0.05)	<0.001***	0.030(0.07)	0.045*	2.700(1.74)	<0.001***
	N1	35(32.7)	0.110(0.21)		0.050(0.06)		0.040(0.11)		4.320(2.78)	
	N2-3	18(16.8)	0.180(0.19)		0.070(0.15)		0.070(0.08)		4.920(3.35)	
Histological grading	Ⅰ	11(10.3)	0.170(0.21)	0.187	0.050(0.09)	0.822	0.080(0.23)	0.109	1.410(2.19)	0.318
	Ⅱ	75(70.1)	0.100(0.12)		0.040(0.04)		0.040(0.07)		1.540(1.04)	
	Ⅲ	21(19.6)	0.100(0.17)		0.030(0.07)		0.030(0.17)		1.980(1.33)	
Vascular invasion	with	12(11.2)	0.135(0.14)	0.366	0.030(0.08)	0.812	0.075(0.07)	0.125	3.605(3.20)	0.988
	non	95(88.8)	0.100(0.14)		0.040(0.05)		0.030(0.10)		3.300(2.42)	
ER	+	86(80.4)	0.100(0.13)	0.561	0.040(0.06)	0.981	0.040(0.07)	0.520	3.145(2.42)	0.103
	-	21(19.6)	0.140(0.27)		0.040(0.08)		0.040(0.15)		4.060(3.64)	
PR	+	74(69.2)	0.100(0.14)	0.594	0.040(0.06)	0.661	0.040(0.09)	0.909	3.145(2.20)	0.277
	-	33(30.8)	0.110(0.18)		0.040(0.06)		0.040(0.11)		4.060(2.94)	
Her-2	+	38(35.5)	0.100(0.15)	0.871	0.040(0.05)	0.791	0.030(0.09)	0.430	3.730(2.89)	0.679
	-	69(64.5)	0.100(0.15)		0.040(0.06)		0.040(0.09)		3.200(2.44)	
Ki-67	+	80(74.8)	0.100(0.15)	0.674	0.040(0.05)	0.324	0.040(0.11)	0.823	3.590(2.73)	0.211
	-	27(25.2)	0.110(0.13)		0.060(0.09)		0.030(0.06)		3.080(1.67)	
AR	+	94(87.9)	0.100(0.14)	0.647	0.040(0.05)	0.348	0.040(0.09)	0.300	3.480(2.49)	0.249
	-	13(12.1)	0.100(0.17)		0.060(0.08)		0.030(0.08)		2.900(1.53)	
Molecular subtype	Luminal A	20(18.7)	0.095(0.15)	0.726	0.050(0.09)	0.927	0.030(0.07)	0.550	3.015(1.18)	0.157
	Luminal B	68(63.6)	0.100(0.13)		0.040(0.05)		0.040(0.09)		3.380(2.59)	
	Her-2 overexpression	12(11.2)	0.060(0.22)		0.035(0.10)		0.025(0.10)		3.730(3.54)	
	Triple-negative expression	7(6.5)	0.150(0.53)		0.040(0.05)		0.110(0.12)		4.810(3.57)	

Clinical Lymph Node Categories: N0: non-metastatic group; N1: metastasis in movable ipsilateral level I, II axillary lymph nodes. N2: metastasis in ipsilateral level I, II axillary lymph nodes fixed to one another (matted); metastasis only in clinically detected ipsilateral internal mammary nodes and in the absence of clinically evident level I, II axillary lymph node metastasis. N3: metastasis in ipsilateral infraclavicular (level III axillary) lymph node (s) with or without level I, II axillary lymph node involvement; metastasis in clinically detected ipsilacral internal mammary lymph node (s) and clinically evident axillary lymph node (s); metastasis in ipsilateral supraclavicular lymph node (s) with or without axillary or internal mammary lymph node involvement

*MDSCs *Myeloid-derived suppressor cells*, PMN-MDSCs *polymorphonuclear MDSCs*, M-MDSCs *monocytic MDSCs*, Tregs *Regulatory T cells*, IQR *Inter quartile range*, AR *Androgen receptor*, PR *Progesterone receptor*, ER *Estrogen receptor*, Her-2 *Human epidermal growth factor receptor 2

*P < 0.05; **P < 0.01; ***P < 0.001

### Clinical data collection

Comprehensive clinical and pathological data were systematically collected from electronic medical records for all breast cancer patients. Data included: age, menopausal status, presence of lymph node metastasis (pathologically confirmed), histological grade (Nottingham grading system), presence of vascular invasion, molecular subtype (defined according to the 2017 St. Gallen International Expert Consensus, and immunohistochemistry (IHC) expression status of ER, progesterone receptor (PR), human epidermal growth factor receptor 2 (Her-2), Ki-67 (proliferation index), and androgen receptor (AR). Her-2 status was determined by IHC (scores 0/1 + considered negative, 3 + considered positive) with fluorescence in situ hybridization (FISH) performed for equivocal (2+) cases. Ki-67 positivity was defined using the institutional standard cutoff (≥ 14%). Detailed demographic and clinicopathological characteristics of the breast cancer group and the control group are presented in Table 1.

### Sample collection and flow cytometry

#### Peripheral blood collection

Fresh peripheral blood samples 2 mL were collected from each participant (patients and controls) into EDTA anticoagulant tubes.

#### Immunophenotyping of MDSCs subsets

Take 100 µL of whole blood into tube, added 10 µL each of eleven antibodies with different fluorescent markers separately, mix well and incubate at room temperature for 15 min, add 800 µL of Erythrocyte lysing solution, mix well and incubate at room temperature for another 15 min, washed twice in 1000 µL phosphate-buffered saline (PBS), then add 500 µL PBS, mix well, to obtain 200,000 nucleated cells for sorting and analysis by flow cytometry.

**Fig. 1:**
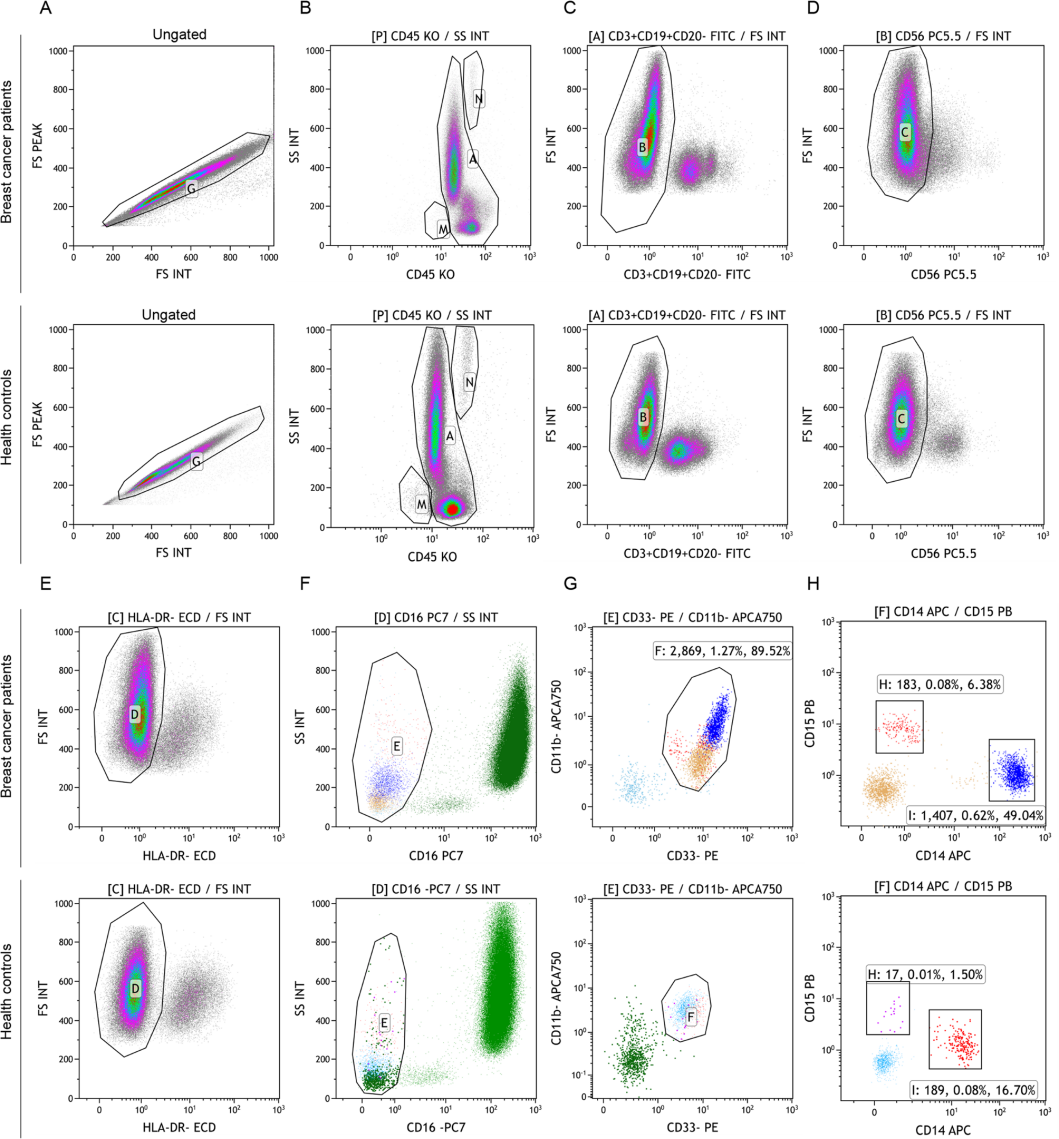
Flow cytometric gating strategy for identification of MDSCs and their subsets in breast cancer patients versus controls. FS INT and FS PEAK are used to establish axes and exclude adherent cells (**A**); The CD45^+^ cell population was identified and basophils and eosinophils were excluded (**B**); The lymphocyte lineage marker negative (LIN-) cell population, i.e. CD3^−^/CD19^−^/CD20^−^, was selected (**C**); The CD56-cell population is identified and CD3^−^CD16^+^CD56^+^ cells were excluded (**D**); The HLA-DR -cell population was identified (**E**); Neutrophils were excluded by selecting the CD16-cell population (**F**); The CD33^+^CD11b^+^ double-positive cell population was identified, which represents all MDSCs (**G**); Monocytic MDSCs were differentiated as the CD15^−^CD14^+^ cell population and granulocytic MDSCs as the CD14^−^CD15^+^ cell population (**H**)

Pathologically activated MDSCs and subsets were identified in freshly isolated PBMCs using a 9-color/11-parameter antibody panel (CD45-KO, CD3-FITC, CD19-FITC, CD20-FITC, CD56-PC5.5, CD16-PC7, HLA-DR-ECD, CD33-PE, CD11b-APC750, CD14-APC, and CD15-PB). The specific steps were as follows:

- Step 1: FS INT and FS PEAK were used to establish axes and exclude adherent cells.
- Step 2: The CD45^+^ cell population was identified and basophils and eosinophils were excluded.
- Step 3:The lymphocyte lineage marker negative (LIN^-^) cell population, i.e. CD3^−^/CD19^−^/CD20^−^, was selected.
- Step 4: The CD56^−^ cell population was identified and CD3^−^CD16^+^CD56^+^ cells were excluded.
- Step 5: The HLA-DR^- ^cell population was identified.
- Step 6: Neutrophils were excluded by selecting the CD16^−^ cell population.
- Step 7: The CD33^+^CD11b^+^ double-positive cell population was identified, which represents all MDSCs.
- Step 8: Monocytic MDSCs were differentiated as the CD15^−^CD14^+^ cell population and granulocytic (Fig. 1A-H).

#### Immunophenotyping of Tregs

**Fig. 2:**
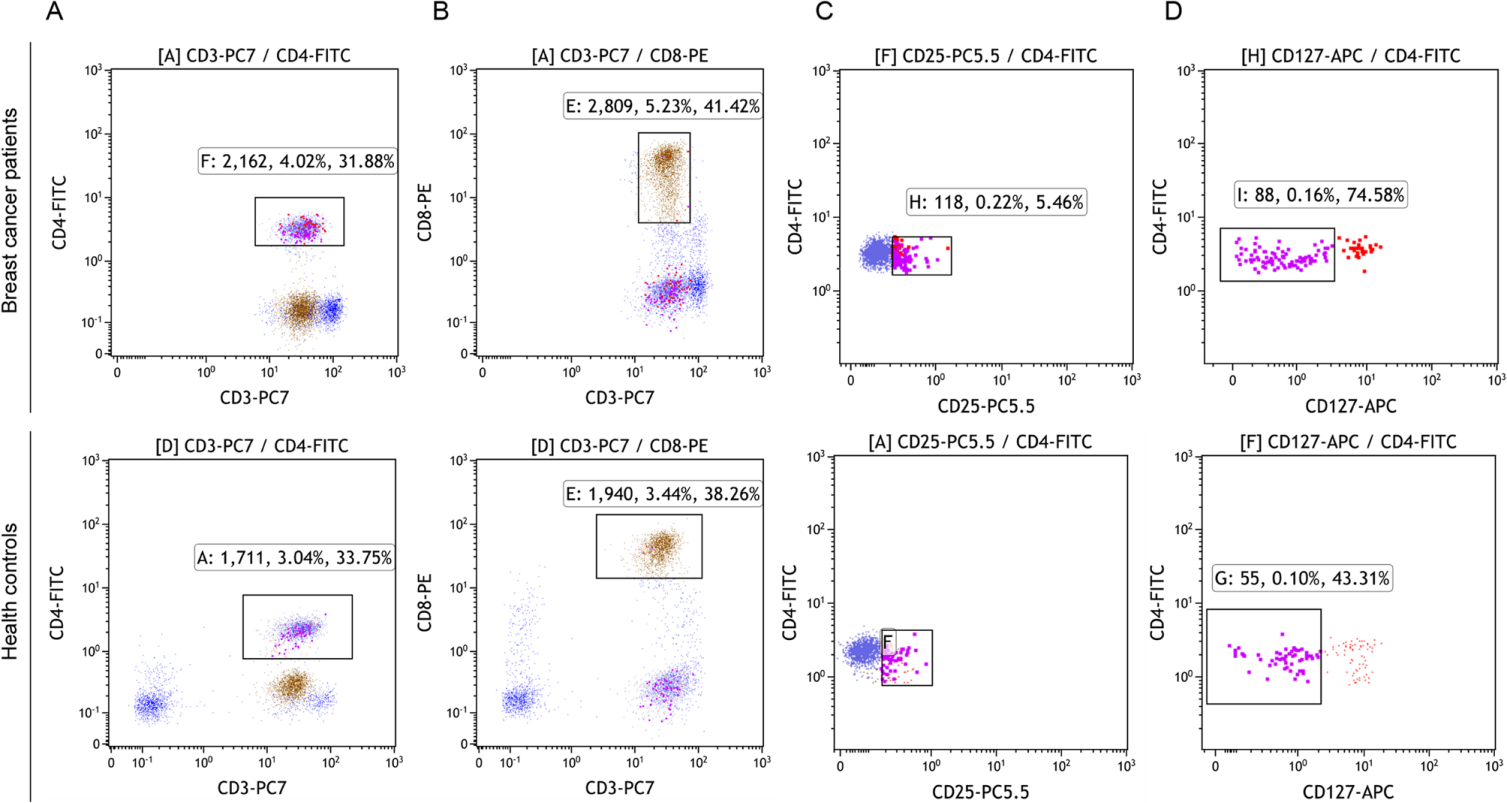
Flow cytometric gating strategy for identification of circulating Tregs in breast cancer patients versus controls. CD3^+^CD4^+^ T cells were identified based on CD3 and CD4 expression (**A**); CD3^+^CD8^+^ T cells were identified based on CD3, CD8 expression (**B**); Within the CD3^+^CD4^+^ T cell population, CD4^+^CD25^+^ cells were identified based on CD25 expression (**C**); Tregs were identified as CD127^-^ cells within the CD4^+^CD25^+^ cell population (**D**)

The method for collecting cells is the same as 2.4.2. Circulating Treg cells were characterized using a 5-color panel (CD3-PC7, CD4-FITC, CD8-PE, CD25-PC5.5, CD127-APC). The specific steps were as follows:

- Step 1: CD3^+^CD4^+^ T cells were identified based on CD3 and CD4 expression.
- Step 2: CD3^+^CD8^+^ T cells were identified based on CD3, CD8 expression.
- Step 3: Within the CD3^+^CD4^+^ T cell population, CD4^+^CD25^+^ cells were identified based on CD25 expression.
- Step 4: Tregs were identified as CD127^−^ cells within the CD4^+^CD25^+^ cell population (Fig. 2A-D).

### Statistical analysis

Statistical analyses were performed using IBM SPSS Statistics (Version 21.0, Armonk, NY). Continuous variables were assessed for normality using the Shapiro-Wilk test. Normally distributed data are presented as mean ± S.E.M (Standard Error of the Mean) and compared between two groups using Student’s *t*-test or between multiple groups using one-way ANOVA with Tukey *post-hoc* testing. Non-normally distributed data are presented as median (inter quartile range) and compared using the Mann-Whitney U test or Kruskal-Wallis test with Dunn’s *post-hoc* test, as appropriate. Categorical variables are presented as frequencies (percentages) and compared using the Chi-square test or Fisher’s exact test. Receiver operating characteristic (ROC) curve analysis was used to evaluate the diagnostic performance of immune cell subsets. Area under the curve (AUC) values were calculated and compared using the DeLong method. A two-sided *P* < 0.05 was considered statistically significant for all analyses.

##  Results

### Elevated MDSCs levels in breast cancer patients

Levels of MDSCs, PMN-MDSCs, and M-MDSCs in peripheral blood were significantly elevated in breast cancer patients versus healthy controls. Total MDSCs showed the most pronounced increase (*P* < 0.001), with PMN-MDSCs (*P* = 0.003) and M-MDSCs (*P* < 0.001) also demonstrating significant elevation (Table 2).

**Table 2:** Comparison of MDSCs, PMN-MDSCs, and M-MDSCs levels in healthy controls versus breast cancer patients. Values expressed as median (IQR)

Groups	n	MDSCs		PMN-MDSCs		M-MDSCs	
		Median(IQR)	*P*	Median(IQR)	*P*	Median(IQR)	*P*
Normal individuals	33	0.03(0.06)	<0.001***	0.02(0.03)	0.003**	0.01(0.03)	<0.001***
Patients with breast	107	0.10(0.14)		0.04(0.06)		0.04(0.09)	

*MDSCs* Myeloid-derived suppressor cells, *PMN-MDSCs *polymorphonuclear MDSCs, *M-MDSCs* monocytic MDSCs, *IQR* Inter quartile range

**P < 0.01; ***P < 0.001

### Levels of MDSCs, PMN-MDSCs, M-MDSCs and Tregs in different clinical subgroups of breast cancer patients

In terms of the levels of MDSCs, PMN-MDSCs, M-MDSCs and Tregs in the peripheral blood of breast cancer patients, no statistically significant correlations were found with age, menopause status, histological grading, vascular invasion, molecular subtypes, ER level, PR level, Her-2 level, Ki-67 level and AR level (all *P* > 0.05). Nevertheless, a notable difference emerged in patients with lymph node metastases, who exhibited significantly higher levels of MDSCs, PMN-MDSCs, and Tregs compared to those without lymph node metastases (*P* < 0.001). Additionally, a statistically significant difference was observed between M-MDSCs and lymph node metastasis (*P* = 0.045) (Table 1).

### Correlation of MDSCs and Tregs levels with lymph node metastasis in breast cancer

Patients with lymph node metastases had significantly higher levels of MDSCs, PMN-MDSCs, M-MDSCs, and Tregs than those without metastases. For MDSCs, significant differences occurred between N0 and N1 (*P* = 0.025) and between N0 and N2-3 (*P* = 0.003), but not between N1 and N2-3 (*P* = 0.739). Regarding PMN-MDSCs, significant differences existed between N0 and N1 (*P* = 0.027) and between N0 and N2-3 (*P* < 0.001), though not between N1 and N2-3 (*P* = 0.475). For M-MDSCs, significant differences were absent in all pairwise comparisons: N0 vs. N1 (*P* = 0.842), N0 vs. N2-3 (*P* = 0.041), and N1 vs. N2-3 (*P* = 0.393). Similarly, Treg levels differed significantly between N0 and N1 (*P* < 0.001) and between N0 and N2-3 (*P* < 0.001), but not between N1 and N2-3 (*P* = 0.410) (Table 3).

**Table 3:** Pairwise comparisons of immune cell levels according to lymph node status (N0, N1, N2–3)

Two-by-two comparison within the group	MDSCs (*P*)	PMN-MDSCs (*P*)	M-MDSCs (*P*)	Tregs (*P*)
N0–N1	0.025*	0.027*	0.842	<0.001***
N0–N2-3	0.003**	<0.001***	0.041*	<0.001***
N1–N2-3	0.739	0.475	0.393	0.41

Clinical Lymph Node Categories: N0: non-metastatic group; N1: metastasis in movable ipsilateral level I, II axillary lymph nodes. N2: metastasis in ipsilateral level I, II axillary lymph nodes fixed to one another (matted); metastasis only in clinically detected ipsilateral internal mammary nodes and in the absence of clinically evident level I, II axillary lymph node metastasis. N3: metastasis in ipsilateral infraclavicular (level III axillary) lymph node (s) with or without level I, II axillary lymph node involvement; metastasis in clinically detected ipsilacral internal mammary lymph node (s) and clinically evident axillary lymph node (s); metastasis in ipsilateral supraclavicular lymph node (s) with or without axillary or internal mammary lymph node involvement

*MDSCs* Myeloid-derived suppressor cells, *PMN-MDSCs* polymorphonuclear MDSCs, *M-MDSCs* monocytic MDSCs, *Tregs* Regulatory T cells

*P < 0.05; **P < 0.01; ***P < 0.001

### ROC curve analysis of MDSCs, PMN-MDSCs, M-MDSCs, and Tregs

**Fig. 3:**
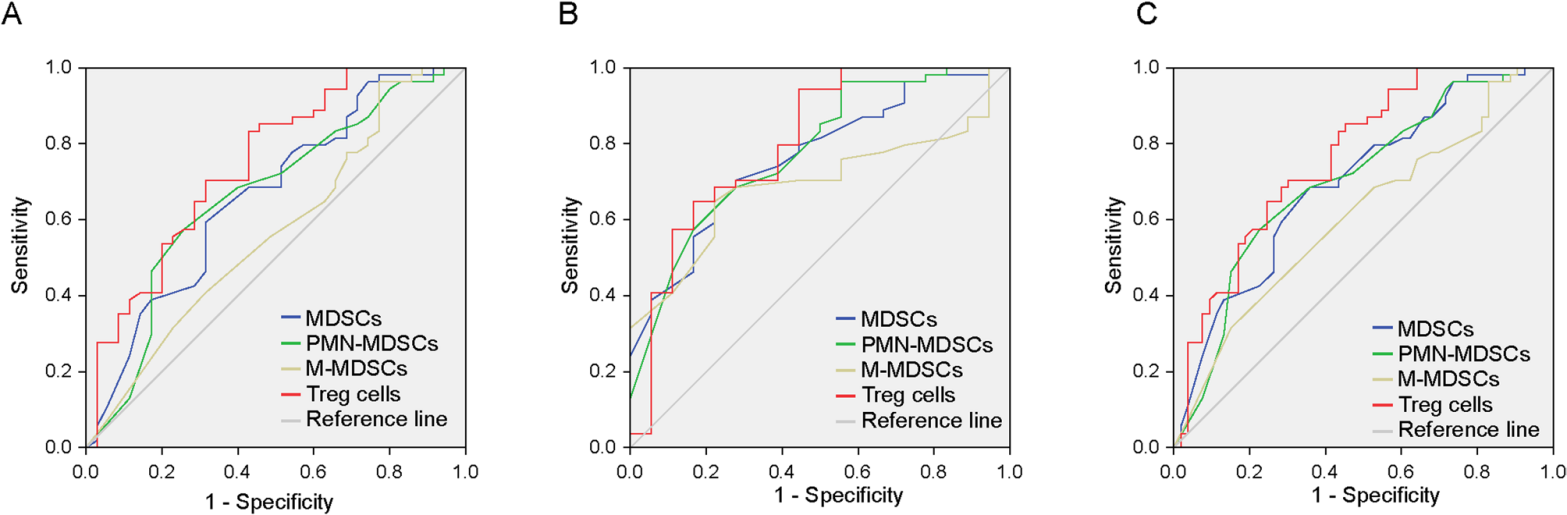
ROC curve analysis of MDSCs, PMN-MDSCs, M-MDSCs, and Tregs comparing N0 with N1, N2–3, and combined metastatic groups. ROC curves for N0 and N1 groups of breast cancer patients (**A**); ROC curves for N0 and N2-3 groups of breast cancer patients (**B**); ROC curves for N0 and N1 + N2-3 groups of breast cancer patients (**C**)

Breast cancer patients were grouped based on lymph node metastasis status. The AUC values for MDSCs and PMN-MDSCs were significantly higher in the N1 metastatic group than in N0 (*P* = 0.009). Similarly, the AUC for Tregs was significantly higher in N1 versus N0 (*P* < 0.001), while no difference was observed for M-MDSCs (*P* = 0.280) (Fig. 3A; Table 4). For the N2-3 metastatic group versus N0, the AUC values of MDSCs, PMN-MDSCs, and Tregs were significantly higher (*P* < 0.001). The AUC of M-MDSCs was also significantly higher in N2-3 than in N0 (*P* = 0.015) (Fig. 3B; Table 4).

**Table 4:** ROC curve analysis of MDSCs, PMN-MDSCs, M-MDSCs, and Tregs for prediction of lymph node metastasis. AUC, sensitivity, specificity, and cut-off values are provided

Variable	Clinical staging	AUC	95%CI	Sensitivity (%)	Specificity (%)	Cut-off	Jordon index	*P*
MDSCs	N0–N1	0.664	0.547 ~ 0.781	0.593	0.686	0.095	0.278	0.009**
	N0–N2-3	0.765	0.646 ~ 0.884	0.685	0.778	0.105	0.463	<0.001***
	N0–N1 + N2-3	0.699	0.600 ~ 0.797	0.685	0.642	0.105	0.327	<0.001***
PMN-MDSCs	N0–N1	0.665	0.547 ~ 0.784	0.574	0.743	0.035	0.317	0.009**
	N0–N2-3	0.779	0.657 ~ 0.902	0.685	0.722	0.045	0.407	<0.001***
	N0–N1 + N2-3	0.704	0.605 ~ 0.803	0.574	0.774	0.035	0.348	<0.001***
M-MDSCs	N0–N1	0.568	0.444 ~ 0.692	0.963	0.229	0.205	0.192	0.280
	N0–N2-3	0.693	0.571 ~ 0.815	0.648	0.778	0.045	0.426	0.015*
	N0–N1 + N2-3	0.610	0.504 ~ 0.717	0.315	0.849	0.015	0.164	0.049*
Tregs	N0–N1	0.748	0.642 ~ 0.853	0.833	0.571	4.065	0.405	<0.001***
	N0–N2-3	0.802	0.673 ~ 0.931	0.944	0.556	4.880	0.500	<0.001***
	N0–N1 + N2-3	0.766	0.677 ~ 0.855	0.648	0.755	3.110	0.403	<0.001***
MDSCs + Tregs	N0–N1	0.771	0.671 ~ 0.871	0.852	0.600	4.175	0.452	<0.001***
	N0–N2-3	0.807	0.679 ~ 0.934	0.648	0.889	3.270	0.537	<0.001***
	N0–N1 + N2-3	0.783	0.697 ~ 0.869	0.648	0.792	3.270	0.441	<0.001***

Clinical Lymph Node Categories: N0: non-metastatic group; N1: metastasis in movable ipsilateral level I, II axillary lymph nodes. N2: metastasis in ipsilateral level I, II axillary lymph nodes fixed to one another (matted); metastasis only in clinically detected ipsilateral internal mammary nodes and in the absence of clinically evident level I, II axillary lymph node metastasis. N3: metastasis in ipsilateral infraclavicular (level III axillary) lymph node (s) with or without level I, II axillary lymph node involvement; metastasis in clinically detected ipsilacral internal mammary lymph node (s) and clinically evident axillary lymph node (s); metastasis in ipsilateral supraclavicular lymph node (s) with or without axillary or internal mammary lymph node involvement

*MDSCs* Myeloid-derived suppressor cells, *PMN-MDSCs* polymorphonuclear MDSCs, *M-MDSCs* monocytic MDSCs, *Tregs* Regulatory T cells, *95% CI* 95% Confidence Interval.

*P < 0.05; **P < 0.01; ***P < 0.001

When comparing the combined N1 and N2-3 metastatic groups to N0, the AUC values for MDSCs, PMN-MDSCs, and Tregs were significantly higher (*P* < 0.001). A significant difference was also observed for M-MDSCs (*P* = 0.049) (Fig. 3C; Table 4).

### Combined detection of MDSCs and Tregs using ROC analysis

**Fig. 4:**
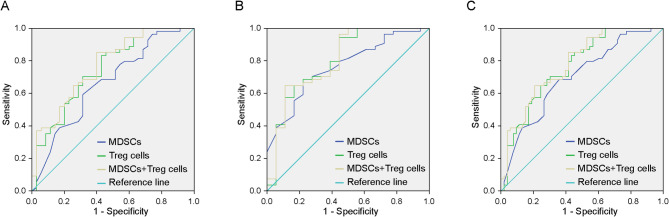
ROC analysis of combined MDSCs + Tregs detection versus individual markers in different lymph node subgroups. Combined ROC curves for N0 and N1 groups of breast cancer patients (**A**); Combined ROC curves for N0 and N2-3 groups of breast cancer patients (**B**); Combined ROC curves for N0 and N1 + N2-3 groups of breast cancer patients (**C**)

ROC analysis compared N0 and N1 groups, revealing significantly higher AUC for combined MDSCs + Tregs detection in the N1 (metastatic) group versus N0(*P* < 0.001) (Fig. 4A; Table 4). Similarly, comparison of N0 and N2-3 groups showed significantly greater AUC for the combined assay (*P* < 0.001) (Fig. 4B; Table 4), consistent with the N0 vs. N1 results. When comparing N0 to the combined N1 + N2-3 metastatic group, the MDSCs + Tregs AUC was significantly higher (*P* < 0.001) (Fig. 4C; Table 4). The combined assay demonstrated statistically significant discrimination across all comparisons (*P* < 0.001).

##  Discussion

Breast cancer remains the leading cause of female cancer mortality and highest-incidence malignancy globally (WHO, 2020) [1, 2]. Current surveillance for recurrence is hampered by the absence of sensitive peripheral blood biomarkers to identify high-risk patients. Critically, tumor progression and regression are governed by host immune status-a relationship central to our investigation of MDSCs and Treg cells.

The study demonstrated that LP-BM5 retrovirus-induced immunodeficiency in mice drives robust expansion of immunosuppressive MDSCs [11]. In this model, highly expressed MDSCs potently suppressed both T- and B-cell immune responses [12]. Koehn et al. further elucidated that MDSCs inhibit systemic immunity through localized mechanisms including amino acid depletion, nitric oxide production, prostaglandin E2 secretion, and reactive oxygen species (ROS) generation [9]. Specifically, MDSCs produce abundant Arg-1 and iNOS, inducing oxidative stress while depleting essential amino acids (e.g., L-arginine, L-cysteine) to suppress T-cell function-ultimately enabling tumor immune escape and systemic immunodeficiency. Consequently, cellular immunity (including T-cell activation and proliferation) becomes severely compromised in advanced cancer patients [13]. MDSCs orchestrate immunosuppression through multiple pathways: inhibiting T/NK cell activation, promoting Th17 differentiation, and facilitating Tregs generation [7]. Concomitantly, they disrupt immune cell differentiation and enhance Tregs proliferation by impeding lymphocyte migration [8]. Notably, Rastad et al. revealed MDSCs also suppress B-cell-mediated immunity via ROS, with M-MDSCs subsets playing a key role in this process [14].

One study defined MDSCs as CD45^+^CD3^−^CD19^−^CD20^−^CD56^−^CD16^−^HLA-DR^−^CD33^+^CD11b^+^ cells. MDSCs are usually classified into two main subtypes: PMN-MDSCs and M-MDSCs. In this study, we analysed MDSCs and their subpopulations (PMN-MDSCs and M-MDSCs) as well as Tregs in the peripheral blood of patients with breast cancer. It is crucial to use standardised isolation procedures, antibody combinations and gating strategies for accurate human MDSCs characterisation, as variations in these methods can affect immunosurveillance outcomes [15]. Following the approach of Apodaca et al., we defined MDSCs as the sum of PMN-MDSCs and M-MDSCs; with subpopulations identified as CD15^−^CD14^+^ (M-MDSCs) and CD14^−^CD15^+^ (PMN-MDSCs). Our results showed that MDSCs, PMN-MDSCs and M-MDSCs were significantly elevated in patients with breast cancer compared to healthy individuals. MDSCs recruited to the tumour tissues and peripheral tissues exhibited different inhibitory functions based on their location, with tumour-derived PMN-MDSCs and M-MDSCs displaying stronger immunosuppressive activities compared to MDSCs derived from peripheral lymphoid organs [16].

MDSCs, immunosuppressive cells derived from the bone marrow, proliferate abnormally in the peripheral blood of patients with breast cancer and are associated with clinical stage and metastatic development [17]. It has been shown [18] that patients with early-stage limited breast cancer often exhibit abnormal proliferation of M-MDSCs compared to those with local recurrence or metastatic breast cancer. Notably, higher MDSCs levels are associated with reduced overall survival in patients with stage IV cancer [19]. To explore the relationship between MDSCs and breast cancer staging, we categorised patients with breast cancer according to molecular subtypes. We observed no significant differences in MDSCs, PMN-MDSCs or M-MDSCs levels among Luminal A, Luminal B, Her-2 overexpression and triple-negative breast cancer patients, suggesting that the peripheral blood level of MDSCs in breast cancer subtypes. This suggests that peripheral blood MDSCs levels in patients with breast cancer with abnormally elevated levels may not be influenced by molecular subtypes. However, MDSCs, PMN-MDSCs and M-MDSCs levels were significantly higher in patients with lymph node metastasis compared to those without. This indicates that peripheral blood MDSCs could serve as a more sensitive marker for diagnosing and determining lymph node metastasis in patients with breast cancer and might aid in clinical diagnosis and prognostic monitoring. Notably, the increase in PMN-MDSCs was more pronounced, with their proliferation surpassing that of M-MDSCs.

MDSCs exert their immunosuppressive functions primarily by directly inhibiting T cell activity and promoting the expansion of Tregs. They impede T cell proliferation and function by depleting essential amino acids such as arginine and tryptophan [20], and the metabolites produced during this process can further disrupt T cell receptor signaling [21]. Additionally, by inducing Tregs production, MDSCs indirectly contribute to a broader immunosuppressive environment within the tumour [22]. Based on the Treg reference intervals established by Lauren et al. [23], our results showed a significant difference in Tregs levels between patients with breast cancer and controls, with higher Tregs expression in patients with breast cancer. Furthermore, we found that Tregs levels were elevated in patients with lymph node metastasis compared to those without. Tregs infiltration within tumours is an independent prognostic factor in breast cancer and has significant predictive value for patient prognosis and late recurrence.

We categorised patients with breast cancer into N0, N1 and N2-3 groups based on lymph node metastasis and compared them using ROC curve analysis. The results demonstrated that the expression levels of MDSCs, PMN-MDSCs, M-MDSCs, Tregs and MDSCs + Tregs tended to increase with advancing disease staging. This suggests that the combined detection of MDSCs and Tregs offers higher sensitivity and diagnostic efficacy for clinical diagnosis and prognosis in breast cancer, significantly enhancing the accuracy of predicting lymph node metastasis.

Collectively, MDSCs and Tregs cooperatively drive tumor immunosuppression, with MDSCs directly potentiating Tregs expansion. This synergy positions MDSCs as critical biomarkers for anti-tumor immunotherapy-facilitating diagnosis, therapeutic stratification, and prognostic monitoring. Critically, elevated peripheral MDSCs (including subsets) and Tregs demonstrate compelling correlation with lymph node metastasis in breast cancer. The combined detection of MDSCs and Tregs emerges as a robust predictor of metastatic involvement, offering significant clinical utility. The detection of lymph node metastasis is a critical factor in guiding clinical surgery and postoperative therapy for breast cancer patients. Our findings, which establish a strong correlation between circulating MDSCs, Tregs, and metastasis, highlight the potential of these cells as non-invasive biomarkers for early detection. This strategy could facilitate individualized precision treatment and improve prognostic assessment. Furthermore, by demonstrating the feasibility of using peripheral blood as a safer and more cost-effective alternative to tissue biopsy, our work paves the way for novel diagnostic approaches. Future studies should focus on elucidating the precise mechanisms of MDSCs-Tregs interactions and evaluating the therapeutic potential of disrupting this axis to improve clinical outcomes.

## Data Availability

No datasets were generated or analysed during the current study.

## Abbreviations

MDSCs: Myeloid-derived suppressor cells
PMN-MDSCs: Polymorphonuclear myeloid-derived suppressor cells
M-MDSCs: Mononuclear myeloid-derived suppressor cells
PBMCs: Peripheral blood mononuclear cells
ER: Estrogen receptor
Tregs: Regulatory T cells
T helper 17: Th17
TME: Tumour micro environment
Her-2: Human epidermal growth factor receptor 2
AR: Androgen receptor
PR: Progesterone receptor
Arg-1: Arginase-1
iNOS: Inducible nitric oxide synthase
ROS: Reactive oxygen species
RNS: reactive nitrogen species
ROC: Receiver response curve
AUC: Area under the curve
